# Health-related quality of life in a cohort of 1070 patients with hypoparathyroidism

**DOI:** 10.1093/ejendo/lvaf169

**Published:** 2025-09-30

**Authors:** Heide Siggelkow, Tamara Vokes, Neil Gittoes, Olulade Ayodele, Maria Luisa Brandi, Felicia Castriota, Michael A. Levine, Carolyn Schaeffer Koziol, Lars Rejnmark, Aliya A. Khan, Pinggao Zhang, Claudio Marelli, John Germak, Michael Mannstadt

**Affiliations:** 1 Department of Trauma, Orthopedic and Reconstructive Surgery, University Medical Center Göttingen, Robert-Koch-Str., 37075 Göttingen, Germany; 2 Center of Endocrine Diseases, Endokrinologikum Göttingen, Von-Siebold-Str, 37075 Göttingen, Germany; 3 Department of Medicine, University of Chicago, Chicago, IL 60637, United States; 4 Centre for Endocrinology, Diabetes and Metabolism, Queen Elizabeth Hospital, Birmingham B15 2TH, United Kingdom; 5 Takeda Development Center Americas, Inc., Cambridge, MA 02142, United States; 6 Fondazione Italiana Ricerca Sulle Malattie dell’Osso (FIRMO Onlus), Florence 50129, Italy; 7 Center for Bone Health and the Division of Endocrinology and Diabetes, Children’s Hospital of Philadelphia and the University of Pennsylvania Perelman School of Medicine, Philadelphia, PA 19104, United States; 8 Takeda Pharmaceuticals U.S.A. Inc., Cambridge, MA 02142, United States; 9 Department of Endocrinology and Internal Medicine, Aarhus University Hospital, 8200 Aarhus, Denmark; 10 Division of Endocrinology and Metabolism, McMaster University, Hamilton, Ontario, L8N 3Z5, Canada; 11 Takeda Pharmaceuticals International AG, 8152 Zurich, Switzerland; 12 Endocrine Unit, Massachusetts General Hospital and Harvard Medical School, Boston, MA 02114, United States

**Keywords:** disease registry, hypoparathyroidism, quality of life, real-world evidence, recombinant human parathyroid hormone (1-84)

## Abstract

**Objective:**

To describe clinical and biochemical characteristics, including health-related quality of life (HRQoL), in patients with chronic hypoparathyroidism (cHypoPT) who were receiving conventional therapy (calcium and active vitamin D), and to compare baseline HRQoL between patients who subsequently received recombinant human parathyroid hormone (1-84) (rhPTH [1-84]) and those who did not.

**Design:**

Cross-sectional analysis of data recorded at enrollment (baseline) from patients with cHypoPT in the PARADIGHM registry before May 31, 2022.

**Methods:**

Eligible patients were aged ≥18 years and receiving conventional therapy at enrollment. Health-related quality of life was measured using the 36-Item Short-Form Health Survey version 2 (SF-36v2), Work Productivity and Activity Impairment Specific Health Problem (WPAI:SHP) questionnaire, and the Hypoparathyroidism Symptom Diary (HypoPT-SD).

**Results:**

Eligible patients (*N* = 1070) were mostly female (80.7%) and White (86.0%). Surgery was the most common cause of cHypoPT (82.2%). Mean serum albumin-adjusted calcium, phosphate, and calcium–phosphate product levels were within the target ranges. Mean (standard deviation [SD]) SF-36v2 physical and mental component scores were 46.3 (10.2) and 47.6 (11.5), respectively (normative scores: 50). Patients who went on to receive rhPTH (1-84) (*n* = 102) had significantly worse baseline HRQoL scores than patients in the conventional therapy group (*n* = 968) for all SF-36v2 and HypoPT-SD parameters and most WPAI parameters. There were no meaningful differences in biochemical parameters between the groups.

**Conclusions:**

Thirty-six-Item Short-Form Health Survey version 2 scores in patients with cHypoPT were lower than the normalized values reported previously for the US population. Patients who were subsequently prescribed rhPTH (1-84) had worse baseline HRQoL scores than those in the conventional therapy group.

## Introduction

Chronic hypoparathyroidism (cHypoPT) is a rare endocrine condition characterized by inappropriately low parathyroid hormone (PTH) levels, which leads to permanent hypocalcemia and hyperphosphatemia.^1,2^ Symptoms reported by patients with cHypoPT include muscle cramps and spasms, paresthesia, fatigue, cognitive decline, anxiety, and depression.^3,4^ Furthermore, patients with cHypoPT have a greater risk of developing renal and cardiovascular disease, cataracts, seizures, nephrocalcinosis/nephrolithiasis, and infections than populations without cHypoPT.^5-8^ Conventional therapy comprising supplemental calcium and active vitamin D analogs does not replace the function of the parathyroid glands and results in inadequate disease control for many patients with cHypoPT.^1,9^

Studies have demonstrated that patients with cHypoPT, the vast majority of whom were receiving conventional therapy, had lower health-related quality of life (HRQoL)^10-12^ and higher rates of healthcare resource utilization (HCRU) than matched populations without cHypoPT or the general population.^3^ Severity of cHypoPT symptoms, inability to work, biochemical abnormalities, and factors related to conventional therapy itself have been suggested to be responsible for the decrease in HRQoL in patients with cHypoPT.^4,13,14^

By contrast, therapy with PTH and PTH analogs has been associated with improvements in HRQoL in patients with cHypoPT.^15-17^ Full-length recombinant human PTH (1-84) (rhPTH [1-84]) was approved as an adjunct to conventional therapy for adults with cHypoPT.^18,19^ Short- and long-term studies have demonstrated that rhPTH (1-84) can improve HRQoL in patients with cHypoPT.^16,17^ In the 24-week REPLACE randomized controlled trial, differences in HRQoL scores between the rhPTH (1-84) and conventional therapy groups were not statistically different; however, scores for the rhPTH (1-84) group improved significantly from baseline for most of the HRQoL parameters measured but did not in the placebo group.^17^ In a long-term open-label study with a follow-up period of over 8 years, patients receiving rhPTH (1-84) reported sustained improvements in HRQoL from baseline.^16^ Similarly, subcutaneously administered palopegteriparatide led to significantly improved scores for the Hypoparathyroidism Patient Experience Scale and the standardized, 36-Item Short-Form Health Survey version 2^®^ (SF-36v2) physical functioning domain compared with placebo, in a 26-week phase 3 study.^15^ Expert clinical guidance recommends that PTH replacement should be considered for patients with HypoPT and poor HRQoL due to inadequate control with conventional therapy.^20^ Nevertheless, there is a lack of real-world evidence regarding the characteristics and HRQoL of patients receiving conventional therapy and rhPTH (1-84) and regarding whether the use of rhPTH (1-84) in routine clinical practice reflects the clinical guidance.

Physicians Advancing Disease Knowledge in Hypoparathyroidism (PARADIGHM) is a multinational, prospective, observational registry designed to collect long-term data from patients with cHypoPT who are receiving conventional therapy, rhPTH (1-84), or both in routine clinical practice.^21^ This registry collects valuable real-world data including HRQoL assessments from the largest reported cohort of patients with cHypoPT to date.

The aims of this analysis were as follows: (1) to describe the characteristics, demographics, biochemical parameters, and HRQoL data of the large population of patients with cHypoPT enrolled in PARADIGHM, (2) to analyze the differences in baseline HRQoL between patients who went on to receive rhPTH (1-84) and those who did not, (3) to assess factors that may influence HRQoL, and (4) to examine factors affecting the decision to prescribe rhPTH (1-84).

## Methods

### Data source

A detailed description of the PARADIGHM registry design has been published previously.^21^ In brief, at its inception in July 2013, PARADIGHM was a disease-state and epidemiology registry. Following a request from the European Medicines Agency, the protocol was amended in November 2017 to allow PARADIGHM to function as a noninterventional postauthorization safety study for rhPTH (1-84). Enrollment ended on January 31, 2023, following the decision by the sponsor, Takeda, to discontinue manufacturing rhPTH (1-84) globally at the end of 2024 owing to unresolved supply issues. Data collection continues for enrolled patients.

PARADIGHM collects and stores anonymized data about patient demographics, clinical characteristics, concomitant medication use, cHypoPT-related signs and symptoms, biochemical parameters, HRQoL, HCRU, and the safety of rhPTH (1-84). The study protocol was approved by each participating site via Independent Ethics Committees, Research Ethics Boards, or Institutional Review Boards (IRBs). For approvals by IRBs, either an institution-specific (27 US sites) or a centralized service provider who was able to review and approve for multiple sites (1 UK, 4 Swedish, and 19 US sites) were used. The IRB of the Mayo Clinic, Rochester, MN, United States, approved the protocol for use at that institution. Written, signed, and approved informed consent was obtained from the patient and/or legally authorized representative/impartial witness before entering PARADIGHM.

### Study design and population

This cross-sectional analysis used data from patients who were enrolled in the PARADIGHM registry up to May 31, 2022 (referred to as the overall population) from 95 centers across 11 countries. Patients enrolled in the registry were included in this analysis if they met the following criteria: were aged 18 years or older, had a diagnosis of cHypoPT (defined as HypoPT with a duration of ≥6 months), and were receiving conventional therapy only (ie, not rhPTH [1-84]) at enrollment. All clinical decisions were made independently by the treating physicians. For this analysis, the safety set included all patients who met the inclusion criteria. The effectiveness set comprised all patients in the safety set excluding those who initiated and discontinued rhPTH (1-84) before enrollment. Patients in the overall population were divided into 2 groups: those who went on to receive rhPTH (1-84) at any time following enrollment, referred to as the rhPTH (1-84) group, and those who continued to receive conventional therapy, referred to as the conventional therapy group.

### Outcome measures

Unless stated otherwise, all outcomes were assessed in the safety set. Health-related quality of life was evaluated using the SF-36v2,^22,23^ and the impact of cHypoPT on daily life was evaluated using the Work Productivity and Activity Impairment Specific Health Problem (WPAI:SHP) questionnaire.^24^

The SF-36v2 generates scores for 8 health domains (physical functioning, role-physical, bodily pain, general health, vitality, social functioning, role-emotional, and mental health) and summary component scores for physical health (physical component summary [PCS]) and mental health (mental component summary [MCS]). Scales are standardized using an algorithm to obtain a score between 0 and 100. A mean score of 50 can be considered as a normative value for all domains and summary scores. Scores above 50 indicate better health status, while scores below 50 indicate worse health status.^22^

The WPAI:SHP provides scores to quantify absenteeism (work time missed), presenteeism (impairment at work), work productivity loss (overall work impairment), and activity impairment. Scores are expressed as percentages, with higher percentages indicating greater impairment.^24^

In addition, we assessed cHypoPT-related symptoms and impacts using the Hypoparathyroidism Symptom Diary (HypoPT-SD), a 13-item, disease-specific patient-reported outcome (PRO) tool developed to evaluate the impact of disease progression and treatment. The Hypoparathyroidism Symptom Diary consists of the HypoPT symptom subscale (items 1-7: item 1 [muscle cramps], item 2 [tingling], item 3 [numbness], item 4 [muscle spasms or twitching], item 5 [feelings of heaviness in the arms], item 6 [physical fatigue], item 7 [slowed or confused thinking], item 8 [anxiety], item 9 [sadness and depression], and items 10-13 [the HypoPT-SD impact subscale]). The tool uses 2 different response scales: items 1-9 use a 5-point ordinal response scale (0 = none, 1 = mild, 2 = moderate, 3 = severe, 4 = very severe) and item 10 (sleep), item 11 (exercise), item 12 (work), and item 13 (relationships) use a 3-point ordinal response scale (0 = not at all, 1 = somewhat, 2 = very much). Higher scores indicate worse symptoms and impacts due to HypoPT.^25-27^

We assessed HCRU by comparing the number of emergency room (ER) visits, cHypoPT-related ER visits, and hospitalizations for any reason that occurred in the 12 months before enrollment. Data regarding the number of ER visits and hospitalizations were obtained using the National Health Interview Survey and the National Health and Nutrition Examination Survey (Adult Version 3.0) administered to all patients at enrollment and during subsequent visits. Prior and concomitant medications were categorized based on the World Health Organization Drug Dictionary medication codes.

Chronic hypoparathyroidism–related biochemical parameters were assessed in the effectiveness set. Data were collected on serum concentrations of albumin-adjusted calcium, phosphorus and magnesium, calcium–phosphorus product, and 24-hour urine calcium excretion. Baseline refers to the first visit at the time of enrollment for all parameters described except biochemical measurements in patients who went on to receive rhPTH (1-84); for these measurements, baseline refers to the last data recorded before initiation of rhPTH (1-84) before or after enrollment.

### Statistical analyses

Continuous variables were summarized using descriptive statistics as the number of patients, mean, standard deviation (SD), and median (range). Categorical variables were summarized as the number and percentage of patients. Unadjusted descriptive statistics and statistics adjusted for missing data are presented for HCRU. Comparative statistical analyses were performed for PRO measure scores only (SF-36v2, WPAI: SHP, and HypoPT-SD). Welch’s *t*-test was used to compare continuous variables between treatment groups, and the *χ*^2^ test was used to compare categorical variables. These tests were not for inferential statistical comparisons between groups and *P*-values indicate trends only. Statistical analyses were performed using SAS^®^ software version 9.4 or later (SAS Institute Inc., Cary, NC, United States). All eligible patients were included in the analyses reported, which were conducted using an observed case approach. Missing data were not imputed, and subgroup analyses by sex and cHypoPT etiology were not prespecified.

## Results

### Demographics, characteristics, and HRQoL of the overall population at baseline

Of the 1302 patients enrolled in the registry, 1070 met the inclusion criteria and were included in the safety analysis set. The majority of patients were female (80.7%) and White (86.0%); the mean (SD) age was 52.3 (15.0) years (Table 1). Surgery was the most common cause of cHypoPT (82.2% of patients), and the mean (SD) duration of disease was 11.4 (11.8) years (Table 1).

In the effectiveness set (*N* = 1046), mean (SD) serum levels of albumin-adjusted calcium, phosphorus, and magnesium were 2.13 (0.26) mmol/L, 1.34 (0.25) mmol/L, and 0.78 (0.08) mmol/L, respectively (target range for albumin-adjusted calcium in patients with cHypoPT: 2.1-2.3 mmol/L^28^; reference range for serum phosphorus: 0.81-1.45 mmol/L^29^; and reference range for serum magnesium: 0.75-1.25 mmol/L).^29^ The mean albumin-adjusted calcium–phosphorus product was 2.98 (0.78) mmol^2^/L^2^ (target threshold <4.4 mmol^2^/L^2^).^28^ Mean (SD) 24-hour urine calcium excretion in females and males were 6.90 (3.56) mmol (276.68 [142.56] mg) and 6.82 (4.71) mmol (273.43 [188.84] mg), respectively. Baseline levels of 24-hour urine calcium excretion above the upper limit of the reference range were reported in 51.5% of female and 38.6% of male patients (upper limit of reference ranges: 6.24 mmol [250 mg]/day for females and 7.49 mmol [300 mg]/day for males [Table 2]).^28^

Muscle cramping (40.1%), paresthesia (36.8%), muscle twitching (26.5%), anxiety (24.2%), brain fog (22.7%), and muscle weakness (22.4%) were reported by patients in the overall analysis population at enrollment. Heaviness in the extremities was reported by 19.3% of patients, and tetany and convulsions or seizures were reported by 15.4% and 5.8% of patients, respectively (Table 3).

Mean (SD) SF-36v2 PCS and MCS scores were 46.3 (10.2) and 47.6 (11.5), respectively. The mean (SD) HypoPT-SD scores for the symptom subscale, impact subscale, anxiety item, and sadness or depression item were 1.0 (0.8), 0.5 (0.6), 0.9 (1.1), and 0.8 (1.0), respectively. Mean (SD) impairment while working due to cHypoPT was 19.0% (24.5), mean (SD) overall work impairment was 21.2% (26.1), mean (SD) activity impairment was 25.3% (29.1), and mean (SD) work time missed was 3.6% (12.2) (Table 4).

### Subgroup analysis

Scores for the SF-36v2 PCS and MCS, and for all parameters measured by the HypoPT-SD, were numerically worse for patients with postsurgical cHypoPT than for patients with nonsurgical cHypoPT. Similarly, SF-36v2 scores and HypoPT-SD scores were numerically worse for female patients than for male patients (Table 5).

### Comparisons between patients with cHypoPT who went on to receive rhPTH (1-84) and those who continued to receive conventional therapy (baseline values)

#### Demographics and clinical characteristics

Of the 1070 eligible patients, 102 (9.5%) were prescribed rhPTH (1-84) at various times following enrollment (the rhPTH [1-84] group) and 968 (90.5%) continued to receive conventional therapy. Demographic and clinical characteristics were similar between the groups (Table 1).

#### Biochemical parameters

There were no clinically meaningful differences in baseline biochemical parameters between the rhPTH (1-84) and conventional therapy group. Mean levels of albumin-adjusted serum calcium, serum phosphate and magnesium, 24-hour urine calcium excretion in male patients, and serum calcium–phosphate product were within the target or reference ranges in both groups (mean [SD] concentrations for rhPTH [1-84] vs conventional therapy group [mmol/L for all measurements except calcium–phosphorus product, which is reported as mmol^2^/L^2^: albumin-corrected serum calcium, 2.12 (0.36) vs 2.14 (0.24); serum phosphorus, 1.35 (0.27) vs 1.34 (0.25); serum magnesium, 0.77 (0.10) vs 0.78 (0.08); and calcium–phosphorus product, 2.95 (0.93) vs 2.98 (0.75) [Table 2]). The mean urine calcium excretion level was above the reference range in female patients. The proportion of patients with baseline 24-hour urine calcium excretion levels above the sex-specific thresholds was higher in the rhPTH (1-84) group than the conventional therapy group for both sexes (Table 2).

#### Signs and symptoms

The proportion of patients reporting cHypoPT-related symptoms at enrollment was numerically higher in the rhPTH (1-84) group than in the conventional therapy group (Table 3).

#### Concomitant medications

At baseline, the use of opioids, antidepressants, anxiolytics, thiazides, and diuretics other than thiazides was numerically higher in the rhPTH (1-84) group than in the conventional therapy group (Table 6). Similarly, the use of native vitamin D and magnesium supplements was more common in the rhPTH (1-84) group than in the conventional therapy group (Table 6).

#### Health-related quality of life

The mean (SD) baseline SF-36v2 PCS and MCS scores were significantly lower (worse) in the rhPTH (1-84) group than in the conventional therapy group (PCS: 42.7 [9.9] vs 46.7 [10.1], *P* = .0002; MCS: 41.5 [12.5] vs 48.2 [11.1], *P* < .0001) (Table 5; Figure 1A and B). Across all SF-36v2 domains, baseline scores were significantly lower (worse) in the rhPTH (1-84) group than in the conventional therapy group (*P* ≤ .0031 in all cases) (Table 5).

#### Impact and symptom burden of cHypoPT

Baseline HypoPT-SD scores for the symptom subscale, impact subscale, anxiety item, and sadness or depression item were significantly higher (worse) in patients who went on to receive rhPTH (1-84) than in those who continued to receive conventional therapy (mean [SD] scores: 1.9 [0.9] vs 0.9 [0.8], *P* < .001; 1.0 [0.5] vs 0.5 [0.5], *P* < .001; 1.6 [1.2] vs 0.8 [1.1], *P* = .0022; and 1.4 [1.0] vs 0.8 [1.0], *P* = .0042) (Table 5; Figure 2).

The mean (SD) WPAI:SHP scores for activity impairment, overall work impairment, and impairment while working were significantly higher (worse) in the rhPTH (1-84) group than in the conventional therapy group (39.1% [32.2] vs 23.8% [28.3], *P* < .0001; 31.4% [29.0] vs 20.2% [25.7], *P* = .0188; and 29.6% [28.5] vs 18.1% [23.9], *P* = .0117, respectively) (Table 5; Figure 3).

#### Subgroup analysis

Among female patients, scores for the SF-36v2 PCS and MCS, and for all parameters measured by the HypoPT-SD, were significantly worse at baseline in the rhPTH (1-84) group than in the conventional therapy group. The same pattern was observed in patients with cHypoPT of surgical origin. In patients with cHypoPT of nonsurgical origin, the MCS score was significantly lower in the rhPTH (1-84) group than in the conventional therapy group, but there was no significant difference in the PCS scores.

Differences between groups in scores for the SF-36v2 PCS and MCS, HypoPT-SD symptom subscale, impact subscale, anxiety, and sadness or depression items were not significant in male patients (Table 6). Differences between groups were not significant in patients with nonsurgical cHypoPT for the HypoPT-SD symptom subscale, anxiety item, or sadness or depression item (Table 6).

#### Healthcare resource utilization

The proportions of patients who required one or more ER visits for any cause and for cHypoPT-related causes in the 12-month period before enrollment were numerically higher in the rhPTH (1-84) group than in the conventional therapy group (53.9% vs 26.7% and 43.1% vs 12.2%, respectively) (Figure 4A and B). In the same period, 37.3% of patients in the rhPTH (1-84) group and 14.0% of patients in the conventional therapy group required hospitalization (Figure 4C). After adjusting for missing data, the proportions of patients in the rhPTH (1-84) and conventional therapy groups who required ER visits, HypoPT-related ER visits, and hospitalizations were 57.3% vs 31.6%, 78.6% vs 45.6%, and 39.6% vs 16.7%, respectively (Figure 4A-C).

## Discussion

This observational, cross-sectional analysis of data from over 1000 patients with cHypoPT is one of the largest studies to date conducted in this rare disease. This cohort had typical characteristics for patients with cHypoPT: the majority were female, and most had postsurgical cHypoPT.

In the overall population at baseline, mean serum levels of albumin-adjusted calcium, phosphate, calcium–phosphate product and magnesium, and 24-hour urine calcium excretion in males were within the target ranges for patients with cHypoPT,^28,29^ whereas 24-hour calcium excretion among female patients was above the threshold for hypercalciuria.^28^ Over half of female patients and almost a third of male patients had 24-hour urine calcium excretion levels above the sex-specific target ranges. Consistent with previous reports, SF-36 scores were lower than normative population values,^12,30,31^ and the symptom burden was considerable. Because the HypoPT-SD is a disease-specific instrument, it has not been tested in the general population; comparisons between patients with cHypoPT and the general population cannot be made.

Comparing baseline characteristics between patients who subsequently went on to receive rhPTH (1-84) treatment and those who did not, we observed remarkable differences in baseline HRQoL and symptom scores, which were worse in the rhPTH (1-84) group. Scores reported for the SF-36v2, HypoPT-SD, and WPAI:SHP indicated that patients who went on to receive rhPTH (1-84) had worse HRQoL, a greater symptom burden, and greater impairment of work and activity than patients in the conventional therapy group. Differences between the 2 groups observed in the generic PRO tool scores were mirrored in the disease-specific HypoPT-SD scoring, which further supports the construct validity of this instrument.

In general, differences between groups in PROs were more pronounced in female patients than in male patients and in postsurgical patients than in nonsurgical patients, although there was a trend toward poorer PROs in the rhPTH (1-84) group compared with the conventional therapy group in the nonsurgical population. The sex-related differences in PROs in this study reflect trends reported in the general population; studies have shown that the prevalence of pain, anxiety, and depression are higher in females than in males.^32-34^ In addition, our study included lower numbers of male patients compared with female patients and nonsurgical patients compared with postsurgical patients, which may have influenced the differences observed between these subgroups.

The differences in PROs between the rhPTH (1-84) and conventional therapy groups were not explained by differences in biochemical parameters relevant to cHypoPT; serum levels of albumin-adjusted calcium, phosphate magnesium, and calcium–phosphate product were similar in both groups. However, the proportion of patients with 24-hour urine calcium excretion above the sex-specific threshold was higher in patients who went on to receive rhPTH (1-84) than in those who remained on conventional therapy only (58.9% vs 49.5% for females and 69.2% vs 31.6% for males).^28,35^ Chronic hypoparathyroidism symptom burden was, however, greater in the rhPTH (1-84) group than in the conventional therapy group, suggesting that inadequate symptom control with conventional therapy could be contributing to both the differences in HRQoL observed between groups and the decision to prescribe rhPTH (1-84).

Consistent with the differences in the HypoPT-SD scores between groups, the symptom burden at enrollment was also worse in the rhPTH (1-84) group. This suggests that the HypoPT-SD tool offers an accurate, quantifiable representation of symptom burden in patients with cHypoPT.

Antidepressant and anxiolytic use were higher in the rhPTH (1-84) group than in the conventional therapy group, which may reflect the differences observed in the SF-36v2 MCS scores and the HypoPT-SD anxiety and sadness or depression single-item scores. The higher use of opioids in the rhPTH (1-84) group suggests that these patients experienced more pain, and the higher use of native vitamin D suggests that a more intensive treatment regimen was required to manage calcium levels. In addition, there was a trend toward higher rates of HCRU in the rhPTH (1-84) group than in the conventional therapy group, which may reflect the higher symptom burden in this group.

### Strengths and limitations

Strengths of this analysis include the large cohort of patients, the formal statistical analysis of HRQoL measures, and the use of a disease-specific PRO tool. The following limitations, however, should be noted: the decision to prescribe rhPTH (1-84) was made by treating physicians, and the reasons behind their decisions were not captured in the registry; furthermore, the prescription of rhPTH (1-84) may have been affected by local availability, reimbursement policies, and/or physician preference; the accuracy of the HRQoL scores reported by patients may be limited by recall bias, although this is likely to be nondifferential (ie, similar in both groups). A small number of patients in the conventional therapy group received teriparatide after enrollment, in which cases, the decision to prescribe may also have been associated with diminished HRQoL. In addition, the proportion of patients who provided HypoPT-SD scores was relatively low, the start and stop timings for concomitant medications were not known, and the impact of hypothyroidism on HRQoL could not be assessed. Similarly, baseline biochemical data were not available for all patients included in this analysis, and data were not adjusted for study center.

## Conclusions

Our analysis of over 1000 patients with cHypoPT who were receiving conventional therapy indicates that, although the mean biochemical parameters were within the acceptable ranges, patient-reported HRQoL as measured by the SF-36v2 was lower than that observed in the general population. Patients who subsequently received rhPTH (1-84) had lower HRQoL and a higher symptom burden compared with patients in the conventional therapy group at enrollment despite similar biochemical parameters. This implies that poor HRQoL may be a relatively independent consideration when physicians initiate rhPTH (1-84), in accordance with the recent clinical recommendations on the use of PTH replacement.

## Figures and Tables

**Figure 1.**
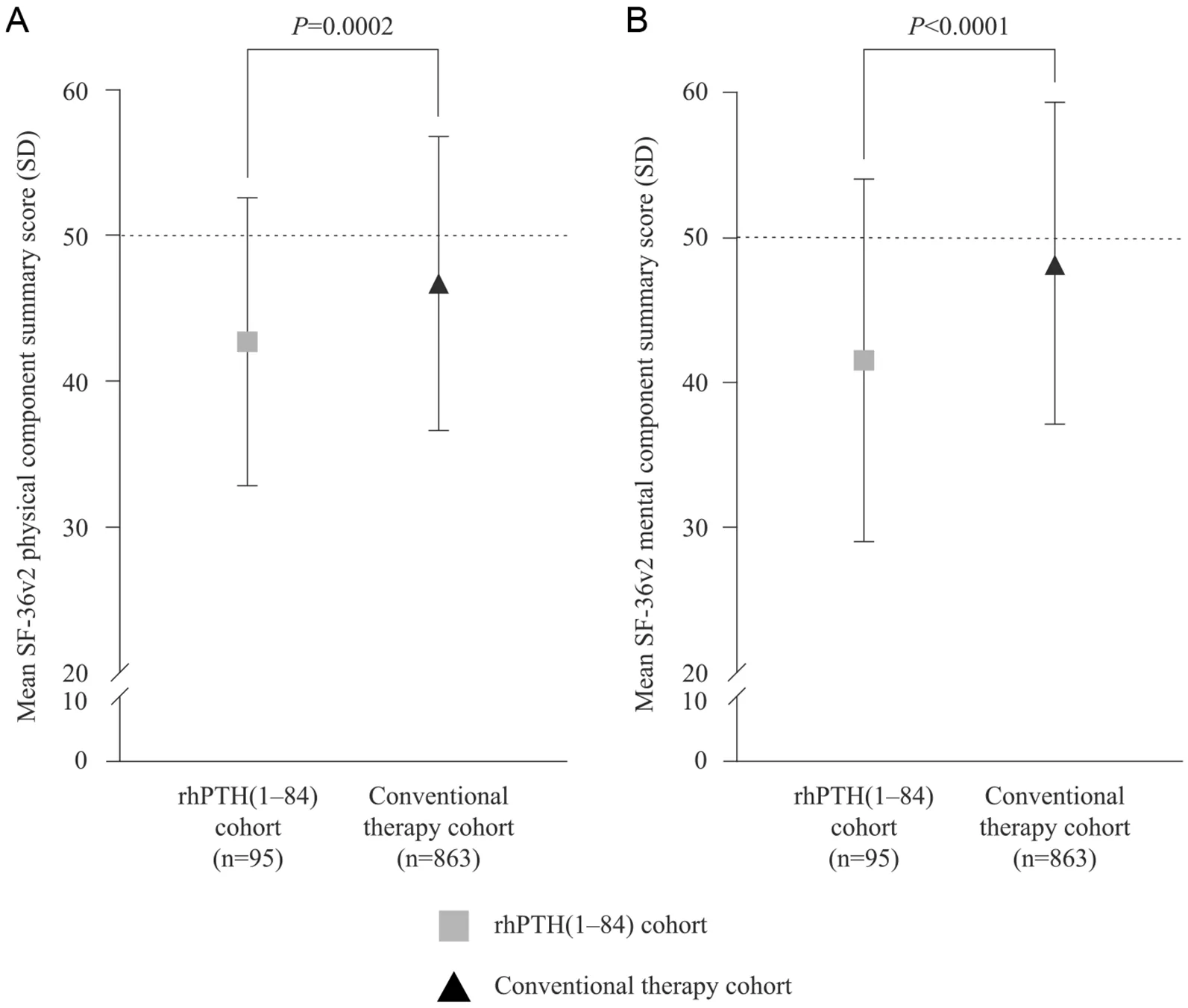
Short-Form 36-Item Health Survey questionnaire version 2 scores (safety set). (A) Baseline physical component summary scores. (B) Baseline mental component summary scores. SF-36 scores range from 0 to 100; higher scores indicate better health status, dotted lines indicate the normative score of 50 (SD = 10), obtained from a US general population.^22^ Abbreviations: rhPTH (1-84), recombinant human parathyroid hormone (1-84); SD, standard deviation; SF-36v2, 36-Item Short-Form Health Survey questionnaire version 2.

**Figure 2.**
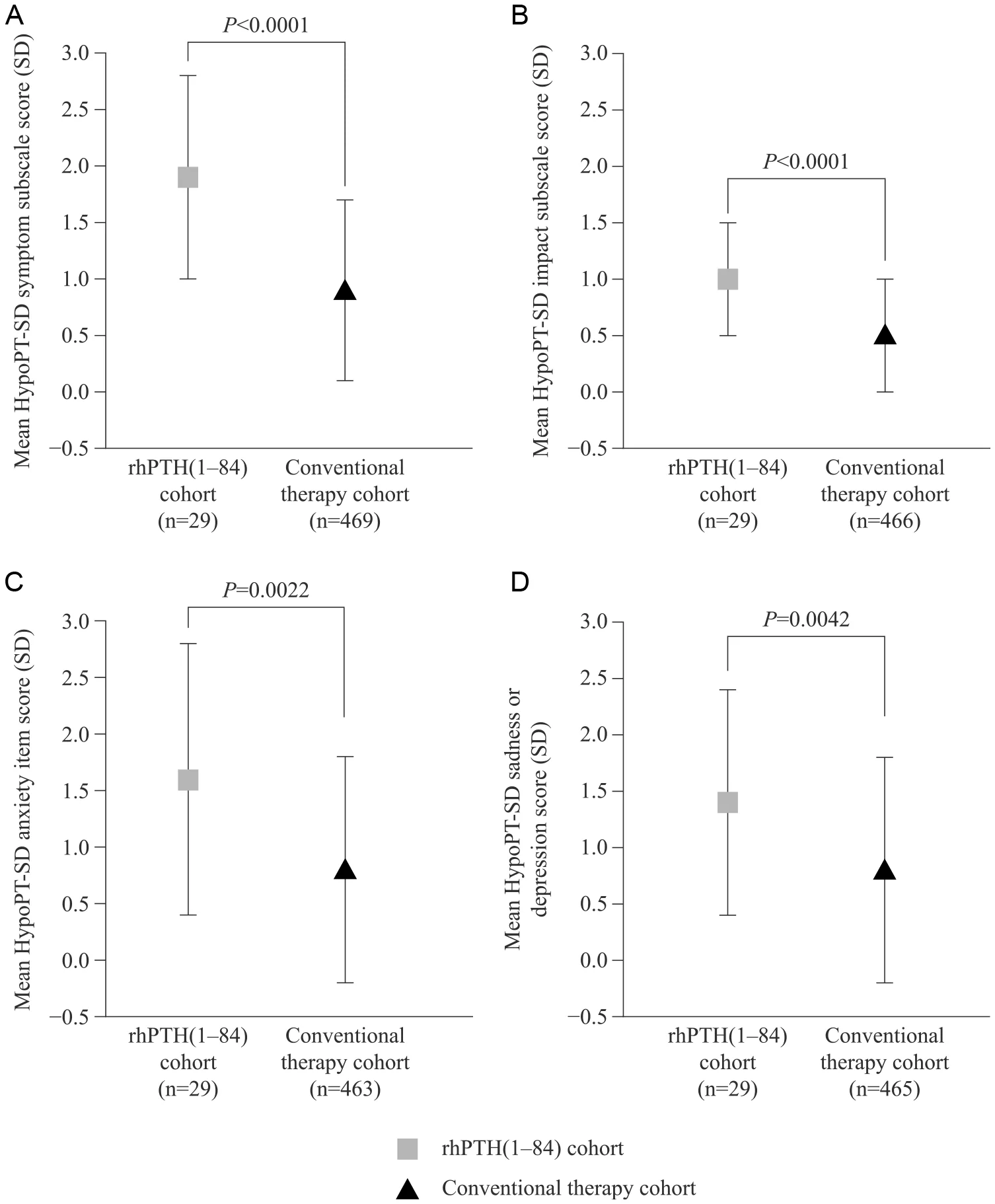
Hypoparathyroidism Symptom Diary scores (safety set). (A) Baseline symptom subscale scores. (B) Baseline impact subscale scores. (C) Baseline anxiety item score. (D) Baseline sadness or depression score. The HypoPT-SD symptom subscale has 7 items scored 0-4, the impact subscale has 4 items scored 0-2, and the anxiety or depression item is scored 0-4. Higher scores indicate worse symptoms or greater impact of HypoPT.^25^ Abbreviations: HypoPT-SD, Hypoparathyroidism Symptom Diary; rhPTH (1-84), recombinant human parathyroid hormone (1-84); SD, standard deviation.

**Figure 3.**
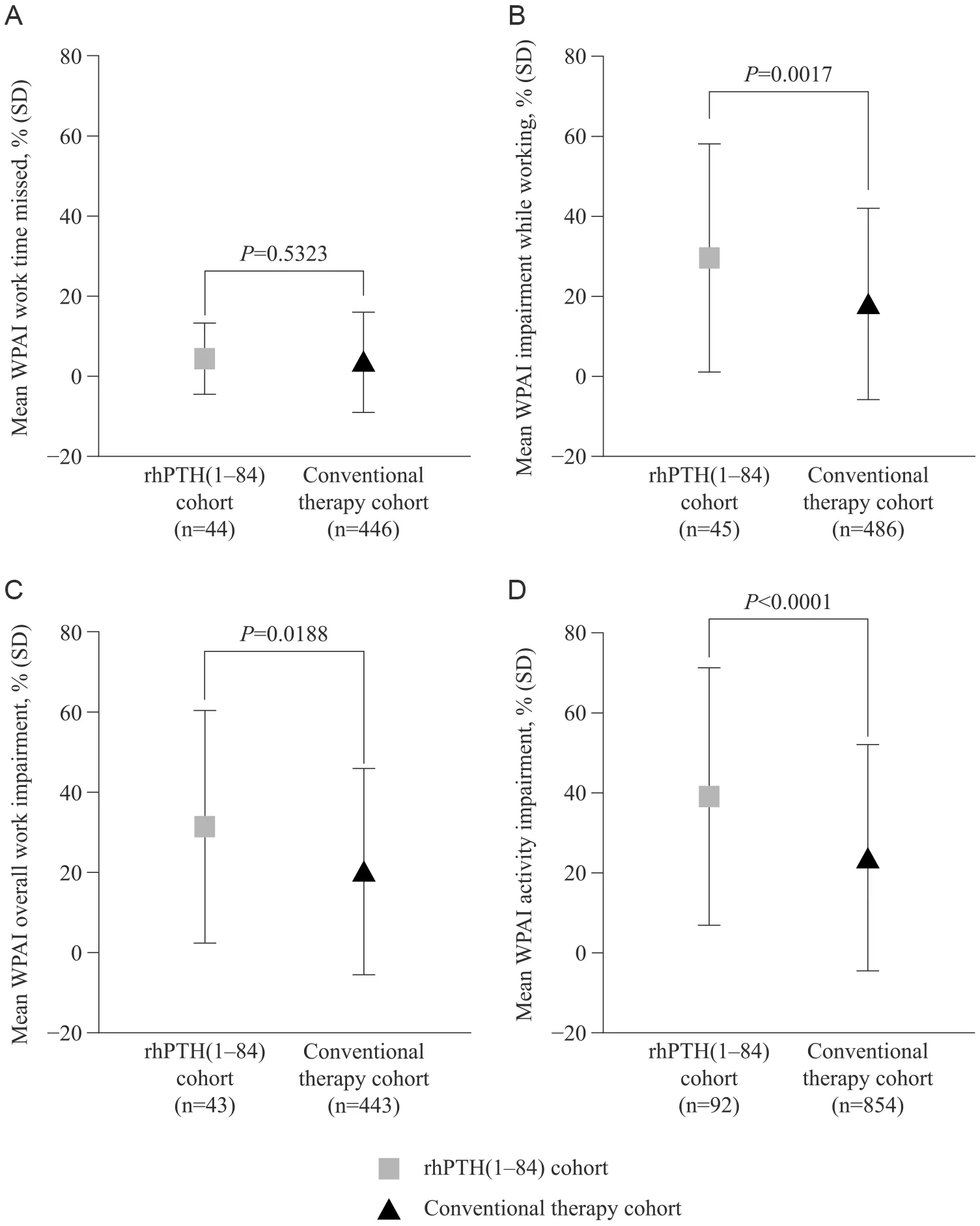
Work productivity and activity impairment specific health problem questionnaire scores (safety set). (A) Work time missed. (B) Impairment while working. (C) Overall work impairment. (D) Activity impairment. WPAI:SHP outcomes are expressed as impairment percentages, with higher percentages indicating greater impairment and less productivity (ie, worse outcomes).^24^ Abbreviations: cHypoPT, chronic hypoparathyroidism; rhPTH (1-84), recombinant human parathyroid hormone (1-84); SD, standard deviation; WPAI:SHP, Work Productivity and Activity Impairment Specific Health Problem questionnaire.

**Figure 4.**
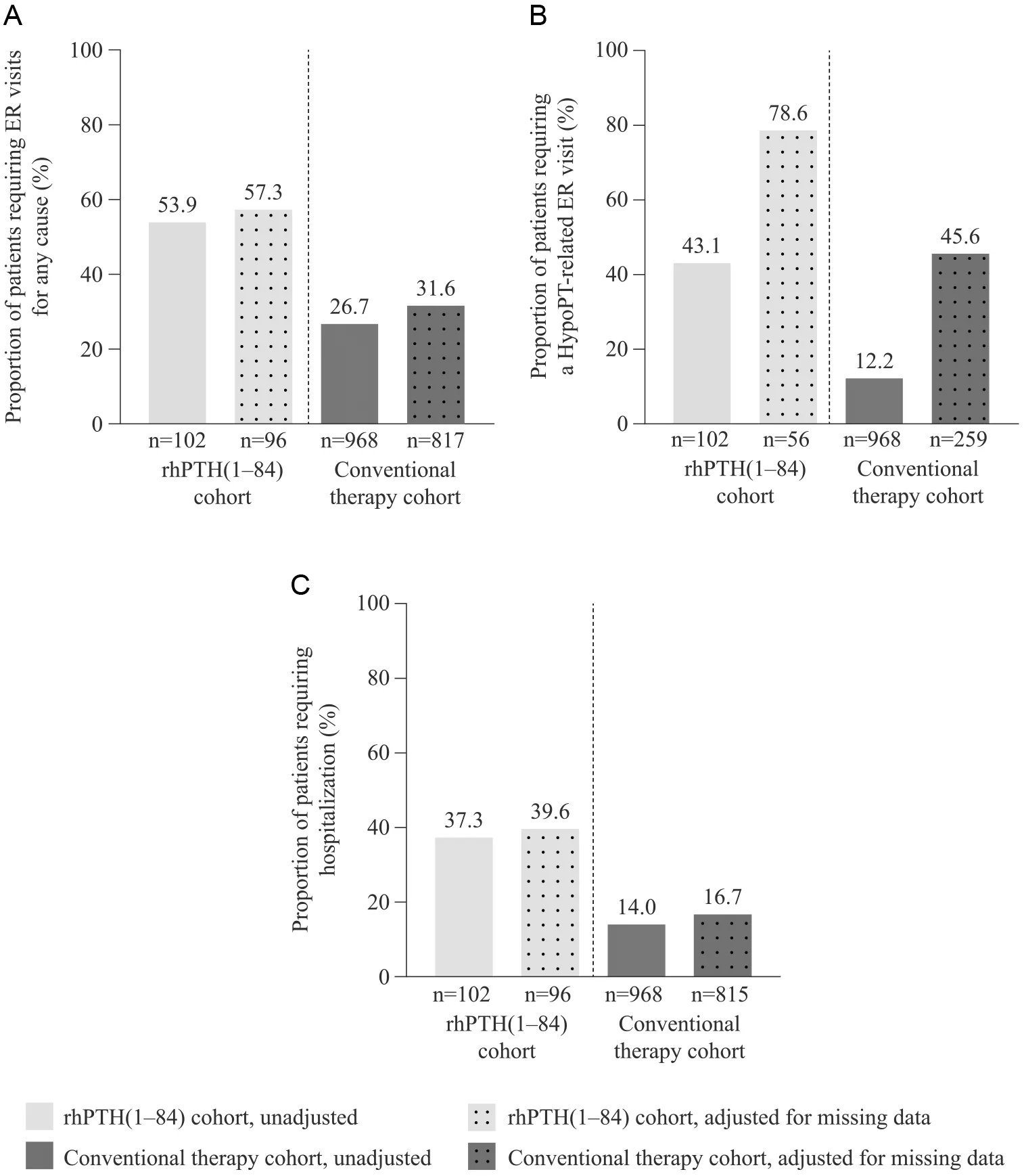
Healthcare resource utilization in the 12-month period before enrollment (safety set).^a^ (A) All emergency room visits. (B) Emergency room visits due to chronic hypoparathyroidism. (C) Hospitalizations. Abbreviations: ER, emergency room; cHypoPT, chronic hypoparathyroidism; rhPTH (1-84), recombinant human parathyroid hormone (1-84). ^a^Formal statistical analyses were not carried out for these parameters.

**Table 1.** Baseline demographics and clinical characteristics (safety set).

Demographic or characteristic	Overall (*N* = 1070)	rhPTH (1-84) (*n* = 102)	Conventional therapy (*n* = 968)
Age			
Years, mean (SD)	52.3 (15.0)	47.6 (12.3)	52.8 (15.1)
Sex			
Female, *n* (%)	863 (80.7)	87 (85.3)	776 (80.2)
Race			
White, *n* (%)	920 (86.0)	90 (88.2)	830 (85.7)
Primary cause of cHypoPT, *n* (%)			
Surgery	880 (82.2)	70 (68.6)	810 (83.7)
Idiopathic	86 (8.0)	13 (12.7)	73 (7.5)
Radiation	1 (0.1)	0 (0.0)	1 (0.1)
Unknown/other/missing	52 (4.9)	11 (10.8)	41 (4.2)
Duration of disease^a^			
Mean (SD), years	11.4 (11.8)	9.9 (10.4)	11.6 (11.9)
Observation period			
Mean (SD), years	3.0 (2.3)	4.3 (2.6)	2.9 (2.2)

Abbreviations: cHypoPT, chronic hypoparathyroidism; rhPTH (1-84), recombinant human parathyroid hormone (1-84); SD, standard deviation.

a Data available for rhPTH (1-84), *n* = 92; conventional therapy, *n* = 941; and overall analysis population, *N* = 1033.

**Table 2.** Baseline biochemical parameters (effectiveness set).

	Overall analysis population (*N* = 1046)		rhPTH (1-84)^a^ (*n* = 102)		Conventional therapy (*n* = 944)	
	*n* ^b^	Measurement	*n* ^b^	Measurement	*n* ^b^	Measurement
Albumin-adjusted serum calcium, mean (SD)^c^						
mmol/L	643	2.13 (0.26)	84	2.12 (0.36)	559	2.14 (0.24)
mg/dL		8.54 (1.04)		8.50 (1.44)		8.58 (0.96)
Serum phosphorus, mean (SD)^c,d^						
mmol/L	691	1.34 (0.25)	84	1.35 (0.27)	607	1.34 (0.25)
mg/dL		4.15 (0.77)		4.18 (0.84)		4.15 (0.77)
Calcium–phosphorus product, mean (SD)^c,d^						
mmol^2^/L^2^	569	2.98 (0.78)	78	2.95 (0.93)	491	2.98 (0.75)
mg^2^/dL^2^		36.95 (9.67)		36.58 (11.53)		36.95 (9.30)
24-hour urine calcium excretion in female patients, mean (SD)^c^						
mmol	247	6.90 (3.56)	31	8.16 (3.97)	216	6.72 (3.47)
mg		276.68 (142.56)		327.17 (159.14)		269.44 (138.93)
24-hour urine calcium excretion in male patients, mean (SD)^c^						
mmol	65	6.82 (4.71)	8	6.13 (2.44)	57	6.92 (4.95)
mg		273.43 (188.84)		245.65 (97.60)		277.33 (198.59)
Proportion of female patients with 24-h urine calcium excretion above upper limit of the reference range^c^						
—	140	51.5%	33	58.9%	107	49.5%
Proportion of male patients with 24-h urine calcium above the upper limit of the reference range^c^						
—	27	38.6%	9	69.2%	18	31.6%
Serum magnesium, mean (SD)^c^						
mmol/L	592	0.78 (0.08)	78	0.77 (0.1)	514	0.78 (0.08)
mg/dL		1.90 (0.19)		1.90 (0.24)		1.90 (0.19)

Target range for patients with cHypoPT: albumin-adjusted serum calcium, 2.1-2.3 mmol/L (8.42-9.22 mg/dL)^28^; calcium–phosphorus product <4.4 mmol^2^/L^2^ (54.56 mg^2^/dL^2^)^28^; normal reference ranges: serum magnesium 0.75-1.25 mmol/L (1.82-3.04 mg/dL); serum phosphorus 0.81-1.45 mmol/L (2.51-4.49 mg/dL)^29^; urine calcium excretion 6.25 mmol (250 mg)/day for women, 7.5 mmol (300 mg)/day for men.^28^

Abbreviations: cHypoPT, chronic hypoparathyroidism; rhPTH (1-84), recombinant human parathyroid hormone 1-84; SD, standard deviation.

a Baseline refers to most recent value before initiation of rhPTH (1-84).

b Number of patients for whom data were available.

c Formal statistical analyses were not carried out for this parameter.

d Concentrations reported as phosphorus, and conversion to mmol/L was based on molecular weight of phosphorus.

**Table 3.** Signs and symptoms of chronic hypoparathyroidism reported by patients at baseline (safety set).

Sign or symptom^a^	Overall analysis population (*N* = 1070)	rhPTH (1-84) (*n* = 102)	Conventional therapy (*n* = 968)
	*n* (%)	*n* (%)	*n* (%)
Anxiety	259 (24.2)	37 (36.3)	222 (22.9)
Brain fog	243 (22.7)	33 (32.4)	210 (21.7)
Convulsions or seizures	62 (5.8)	15 (14.7)	47 (4.9)
Heaviness in extremities	207 (19.3)	28 (27.5)	179 (18.5)
Muscle cramping	429 (40.1)	47 (46.1)	382 (39.5)
Muscle twitching	284 (26.5)	47 (46.1)	237 (24.5)
Muscle weakness	240 (22.4)	32 (31.4)	208 (21.5)
Paresthesia	394 (36.8)	60 (58.8)	334 (34.5)
Tetany	165 (15.4)	29 (28.4)	136 (14.0)

Abbreviations: cHypoPT, chronic hypoparathyroidism; rhPTH (1-84), recombinant human parathyroid hormone 1-84.

a Formal statistical analyses were not carried out for this parameter.

**Table 4.** Patient-reported outcome measure scores recorded at baseline (safety set).

PRO instrument	Overall analysis population (*N* = 1070)		rhPTH (1-84) (*n* = 102)		Conventional therapy (*n* = 968)		*P* value, rhPTH (1-84) vs conventional therapy
	*n* ^a^	Score (SD)	*n* ^a^	Score (SD)	*n* ^a^	Score (SD)	
SF-36v2 mean scores (SD)							
Physical component summary	958	46.3 (10.2)	95	42.7 (9.9)	863	46.7 (10.1)	.0002^b^
Mental component summary	958	47.6 (11.5)	95	41.5 (12.5)	863	48.2 (11.1)	<.0001^b^
Physical functioning	989	47.2 (9.9)	97	43.2 (11.0)	892	47.7 (9.7)	.0002^b^
Role-physical	995	46.1 (10.7)	98	41.7 (11.1)	897	46.6 (10.5)	.0001^b^
Bodily pain	999	46.8 (11.1)	99	43.8 (10.4)	900	47.1 (11.1)	.0031^b^
General health	995	45.5 (11.4)	97	40.2 (10.5)	898	46.1 (11.4)	<.0001^b^
Vitality	995	47.0 (11.1)	97	42.3 (10.8)	898	47.5 (11.0)	<.0001^b^
Social functioning	993	46.5 (11.0)	97	41.1 (11.1)	896	47.1 (10.8)	<.0001^b^
Role-emotional	997	46.8 (11.5)	98	40.5 (14.2)	899	47.5 (10.9)	<.0001^b^
Mental health	992	48.2 (10.8)	96	43.3 (11.8)	896	48.7 (10.6)	<.0001^b^
HypoPT-SD mean scores (SD)							
Symptom subscale	498	1.0 (0.8)	29	1.9 (0.9)	469	0.9 (0.8)	<.0001^b^
Impact subscale	495	0.5 (0.6)	29	1.0 (0.5)	466	0.5 (0.5)	<.0001^b^
Anxiety item score	492	0.9 (1.1)	29	1.6 (1.2)	463	0.8 (1.1)	.0022^b^
Sadness or depression item score	494	0.8 (1.0)	29	1.4 (1.0)	465	0.8 (1.0)	.0042^b^
WPAI:SHP: mean scores (SD)							
Activity impairment	946	25.3 (29.1)	92	39.1 (32.2)	854	23.8 (28.3)	<.0001^b^
Overall work impairment	486	21.2 (26.1)	43	31.4 (29.0)	443	20.2 (25.7)	.0188^b^
Impairment while working	531	19.0 (24.5)	45	29.6 (28.5)	486	18.1 (23.9)	.0117^b^
Work time missed	490	3.6 (12.2)	44	4.4 (8.9)	446	3.5 (12.5)	.5323

Abbreviations: HypoPT-SD, Hypoparathyroidism Symptom Diary; PRO, patient-reported outcome; rhPTH (1-84), recombinant human parathyroid hormone 1-84; SD, standard deviation; SF-36v2, 36-Item Short-Form Health Survey questionnaire version 2; WPAI:SHP, Work Productivity and Activity Impairment Specific Health Problem questionnaire.

a Number of patients for whom data were available.

b Statistically significant *P*-values (<.05).

**Table 5.** Key baseline patient-reported outcome measures stratified by chronic hypoparathyroidism etiology and sex (safety set).

PRO instrument	Overall analysis population (*N* = 1070)		rhPTH (1-84) (*n* = 102)		Conventional therapy (*n* = 968)		*P* value, rhPTH (1-84) vs conventional therapy
	*n* ^a^	Score (SD)	*n* ^a^	Score (SD)	*n* ^a^	Score (SD)	
SF-36v2 PCS mean score (SD)							
Postsurgical	798	45.6 (10.2)	66	40.0 (9.3)	732	46.1 (10.1)	<.0001^b^
Nonsurgical	160	49.9 (9.5)	29	48.7 (8.5)	131	50.2 (9.7)	.4222
Female	781	45.8 (10.2)	83	42.0 (9.8)	698	46.2 (10.1)	.0003^b^
Male	177	48.6 (9.9)	12	47.0 (9.7)	165	48.7 (10.0)	.5814
SF-36v2 MCS mean score (SD)							
Postsurgical	798	47.4 (11.3)	66	40.6 (11.9)	732	48.0 (11.1)	<.0001^b^
Nonsurgical	160	48.3 (12.1)	29	43.4 (14.0)	131	49.4 (11.4)	.0381^b^
Female	781	46.9 (11.6)	83	40.4 (12.7)	698	47.6 (11.2)	<.0001^b^
Male	177	50.8 (10.4)	12	48.6 (9.4)	165	50.9 (10.4)	.4237
HypoPT-SD mean score (SD)							
Symptom subscale							
Postsurgical	430	1.0 (0.8)	23	1.9 (0.8)	407	1.0 (0.8)	<.0001^b^
Nonsurgical	68	0.9 (0.8)	6	1.7 (1.0)	62	0.8 (0.8)	.0735
Female	409	1.1 (0.8)	25	2.0 (0.7)	384	1.0 (0.8)	.0000^b^
Male	89	0.7 (0.7)	4	1.1 (1.3)	85	0.7 (0.7)	.5521
Impact subscale							
Postsurgical	427	0.5 (0.6)	23	1.0 (0.5)	404	0.5 (0.6)	.0002^b^
Nonsurgical	68	0.4 (0.5)	6	1.1 (0.6)	62	0.3 (0.4)	.0212^b^
Female	406	0.6 (0.6)	25	1.1 (0.5)	381	0.5 (0.6)	.0000^b^
Male	89	0.3 (0.5)	4	0.9 (0.8)	85	0.3 (0.5)	.1936
Anxiety item score							
Postsurgical	424	0.9 (1.1)	23	1.5 (1.2)	401	0.9 (1.1)	.0163^b^
Nonsurgical	68	0.8 (1.1)	6	2.0 (1.4)	62	0.7 (1.0)	.0707
Female	405	1.0 (1.1)	25	1.8 (1.2)	380	0.9 (1.1)	.0021^b^
Male	87	0.6 (0.9)	4	0.8 (1.0)	83	0.5 (0.9)	.6976
Sadness or depression item score							
Postsurgical	426	0.9 (1.0)	23	1.3 (0.9)	403	0.8 (1.0)	.0394^b^
Nonsurgical	68	0.8 (1.0)	6	2.0 (1.3)	62	0.7 (0.9)	.0521
Female	406	0.9 (1.1)	25	1.5 (1.0)	381	0.9 (1.1)	.0044^b^
Male	88	0.6 (0.9)	4	1.0 (1.4)	84	0.6 (0.9)	.6089

Abbreviations: cHypoPT, chronic hypoparathyroidism; HypoPT-SD, Hypoparathyroidism Symptom Diary; MCS, mental component summary, PCS, physical component summary; PRO, patient-reported outcome; rhPTH (1-84), recombinant human parathyroid hormone (1-84); SD, standard deviation; SF-36v2, 36-Item Short-Form Health questionnaire version 2.

a Number of patients for whom data were available.

b Statistically significant *P*-values (<.05).

**Table 6.** Baseline prior and concomitant medications (safety set).^a,b^

	Overall analysis population (*N* = 1070)	rhPTH (1-84) (*n* = 102)	Conventional therapy (*n* = 968)
		
	*n* (%)	*n* (%)	*n* (%)
Vitamin D, vitamin D analogs, calcium, magnesium			
Native vitamin D	615 (57.5)	83 (81.3)	532 (55.0)
Calcitriol or alfacalcidol	971 (90.7)	94 (92.2)	877 (90.6)
Calcium	909 (85.0)	88 (86.3)	821 (84.8)
Magnesium	363 (33.9)	58 (56.9)	305 (31.5)
Hormones			
Thyroid hormone	856 (80.0)	75 (73.5)	781 (80.7)
Teriparatide^c^	38 (3.6)	13 (12.7)	25 (2.6)
Thiazides			
Hydrochlorothiazide	174 (16.3)	25 (24.5)	149 (15.4)
Antidepressants	243 (22.7)	44 (43.1)	199 (20.6)
Antihypertensive	223 (20.8)	25 (24.5)	198 (20.5)
Anxiolytics	124 (11.6)	22 (21.6)	102 (10.5)
Diuretics other than thiazides	156 (14.6)	27 (26.5)	129 (13.3)
Opium alkaloids	129 (12.1)	23 (22.5)	106 (11.0)
Platelet aggregation inhibitors	154 (14.4)	13 (12.7)	141 (14.6)
Multivitamins	1038 (97.0)	100 (98.0)	938 (96.9)

Abbreviations: rhPTH (1-84), recombinant human parathyroid hormone 1-84.

a Used by ≥10% of patients.

b Formal statistical analyses were not carried out for this parameter.

c Patients started teriparatide after the US recall of rhPTH (1-84) in 2019 and during the follow-up period.

## Data Availability

The dataset supporting the conclusions of this article is included within the article. Restrictions apply to the availability of data generated or analyzed during this study to preserve patient confidentiality or because they were used under license. On request, the corresponding author will detail the restrictions and any conditions under which access to some data may be provided.

## Significance

Despite receiving conventional therapy with oral calcium and active vitamin D, patients with chronic hypoparathyroidism (cHypoPT) have lower health-related quality of life (HRQoL) than healthy individuals. This analysis of data from a registry of over 1000 patients with cHypoPT receiving conventional therapy suggests that, although serum calcium levels were within the generally accepted target range, HRQoL remained lower than the previously reported normalized values for the US general population. This study also found that patients who subsequently received recombinant parathyroid hormone (1-84) (rhPTH [1-84]) had significantly lower baseline HRQoL scores and a higher symptom burden than patients who continued to receive conventional therapy, suggesting that HRQoL may influence the decision to initiate rhPTH (1-84).
